# Seroprevalence of selected viral pathogens in pigs reared in organized farms of Meghalaya from 2014 to 16

**DOI:** 10.14202/vetworld.2018.42-47

**Published:** 2018-01-20

**Authors:** Priyanka Mukherjee, Amarjit Karam, Uttam Singh, Amit Kumar Chakraborty, Surmani Huidrom, Arnab Sen, Indu Sharma

**Affiliations:** 1 Division of Animal Health, Indian Council of Agricultural Research (ICAR) Research Complex for North Eastern Hill Region, Umiam - 793 103, Meghalaya, India; 2 Department of Microbiology, Assam University, Silchar - 788 011, Assam, India

**Keywords:** antibody, classical swine fever virus, enzyme-linked immunosorbent assay, Meghalaya, *Porcine circovirus*, Porcine respiratory and reproductive syndrome virus, seroprevalence, virus

## Abstract

**Aim:**

A pilot study was carried out to find out the seroprevalence of Porcine circovirus 2 (PCV2), classical swine fever virus (CSFV), and Porcine respiratory and reproductive syndrome virus (PRRS) in pig population of Meghalaya.

**Materials and Methods:**

Serum samples were collected from piglets of 40–45 days age group, growers, and sows reared under organized and unorganized management in 11 districts of Meghalaya situated in the Khasi, Jaintia, and Garo hills divisions in the time period of 2014-2016 from apparently healthy and suspected pigs. Seroprevalence of PCV2, CSFV, and PRRS specific antibodies was detected by enzyme-linked immunosorbent assay (ELISA).

**Results:**

A total of 1899 serum samples were collected and screened using antibody ELISA kits specific for PCV2, CSFV, and PRRS. The highest antibody prevalence during the selected time periods was detected for PCV2 (80.8% in 2014, 79.1% in 2015, and 96.2% in 2016) followed by CSFV (76.4% in 2014, 66.09% in 2015, and 25.5% in 2016) and PRRS (2.8% in 2014, 2.7% in 2015, and 3.62% in 2016). The result indicates high seroprevalence for PCV2, which can be considered as an inducement factor due to the immunosuppressive nature of the virus, for animals being susceptible to other pathogens in farms where airborne transmission of PCV2 and postweaning multisystemic wasting syndrome among animals reared in close pens can be a major possibility.

**Conclusions:**

The data from this study indicates ubiquitous prevalence of PCV2 antibodies in the farm animals along with the endemic presence of swine fever and emergence of PRRS in an organized farm. There are few reports regarding PCV2 infections/outbreaks in pigs associated with reproductive failure from northern and southern part of India, but till date, there are no reports regarding concomitant infection of CSFV and PCV2 from India. Considerable high seropositivity of PCV2 indicates the need for high impact hygiene practice in farms, routine seromonitoring and implementation the vaccination program. To the author’s best knowledge, this is the first documented report on the seroprevalence of PCV2, CSFV, and PRRS from pig population of Meghalaya.

## Introduction

Virus coinfection has become a common phenomenon involving viruses of same or different type or closely related species. Apart from homologous viruses, heterologous viruses form hybrid viruses [1-4] resulting in persistent infection. Porcine circovirus 2 (PCV2) being immunosuppressive in nature was found persistently coinfected with other viruses such as Porcine Parvovirus [5-7], classical swine fever virus (CSFV) [8,9], and Porcine respiratory and reproductive syndrome (PRRS) [10-12] resulting in the outbreak outbreak and pig mortality. Thus, coinfection can be considered as a serious threat to animal husbandry as well as public health.

The causative agent of the postweaning postweaning multisystemic wasting syndrome (PWMS) is PCV2, a member of Circoviridae family of genus *Circovirus*, is a non-enveloped, single-stranded, circular DNA genome. PCV 2 infection does not necessarily imply disease [13,14] and clinical signs are non-specific and variable. Usually, a sudden increase in the mortality rate in the early fattening stage is observed [15], and affected pigs show wasting with or without a respiratory sign, diarrhea, and paleness of the skin and icterus. CSFV or hog cholera is a highly contagious disease and the most important viral disease of domestic pigs, economically affecting various parts of the world [16]. It is characterized as a hemorrhagic fever in its acute form leading to chronic and clinically inapparent form [17,18]. The causative virus is a member of genus *Pestivirus* of the family Flaviviridae, is a small, enveloped virus with a non-segmented, single-stranded positive RNA genome. PRRSV belongs to Arteriviridae family within the genus *Arterivirus*, is an enveloped virus with a linear positive-stranded RNA genome. The virus causes abortions in late pregnancy, stillborn or weak piglets, general reproductive failure, respiratory disease and high death rates in suckling, and weaned pigs [19]. All the three viruses are remarkably adapted to its natural host and exclusively infect pig monocyte and macrophages.

North East India contributes about 38.05% of the total pig population of India where Meghalaya contributes 5.28% of the total pig population of this region (Livestock Census Report-2012, Government of India). Livestock forms an important component of the mixed farming system in this area mainly for two reasons; first due to the preference of meat by the majority in their diets and second the difficult terrain for large-scale crop production. To meet the high demand, the state draws on supplies from outside the state [20].

The rapid development of pig industry in Meghalaya accompanies many CSFV outbreaks in this region [21-23]. Pigs are susceptible to infection that may affect their health and productivity. Although the occurrence of some viral disease agents in pigs has been documented [5,21-24] these studies are mostly based on limited sample size. Thus, it becomes important to expand and update this data especially when we consider emerging diseases. The main objective of this study was to determine antibody prevalence of selected viral pathogens in pigs in organized farms of Meghalaya during the period of April 2014 to October 2016 and draw a conclusive analysis of vaccination profile for CSFV that is limited in some organized farms only.

## Materials and Methods

### Ethical approval

As per Committee for the Purpose of Control and Supervision of Experiments on Animals guidelines, a study involving clinical samples does not require the approval of Institute Animal Ethics Committee. However, the samples collected for the present study followed standard sample collection methods without any harm or stress to the animals.

### Study area and data collection

**Figure-1:**
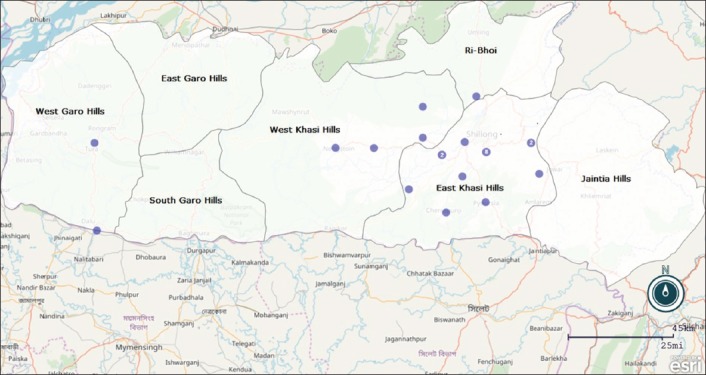
Map of Meghalaya indicating the location of the pig farms from where samples were collected during the period of 2014-2016. The numbers in the dots represent the number of farms located in that area.

Serum samples were collected from piglets age ranging from 40 to 45 days, growers and sows associated with piglets from different organized farms of Meghalaya, namely: Nongpyiur Farm (EKH), Pynursla Farm (EKH), Mairang Farm (WKH), Mawryngkneng farm (JH), Kyrdemkulai farm (Ri Bhoi), Nongstoin farm (WKH), and Lumkhudung (JH) (Figure-1).

Samples were collected from all the organized farms of the three hills division of Meghalaya. The geographical map of Meghalaya indicating the organized farms located in this region depicts that most of the farms where sample collection was done were mainly located in East Khasi Hills, West Khasi Hills, and Ri Bhoi District; countable in Garo and Jaintia Hills. The organized farms of this region have a vaccination history against CSFV till 2011 but the unavailability and shortfall of lapinized CSFV vaccine, the practice of vaccination is limited to few farms where 100% successful vaccination was not recorded.

Samples were collected from the Disease Surveillance Programme throughout the year in the bimonthly interval in 2014 (n=500), 2015(n=292), and 2016 (n=1106) for regular diagnosis of viral pathogens. The animal vaccination history was not recorded but as such in Meghalaya PCV-2, and PRRS vaccination is not being carried out, but some of the farms used to perform CSF vaccination, where successful vaccination outcome was not recorded. These farms were selected on the basis of the large concentration on pig production and high density. Blood samples were collected from intracardiac in piglets and ear vein in older pigs. Blood samples were collected in sterile vacutainer tubes without ethylenediaminetetraacetic acid. Serum was separated and stored at −20°C before the examination.

### Serology

Serum samples were analyzed by commercially available enzyme-linked immunosorbent assays (ELISA) according to the manufacturer’s instructions. Results obtained were expressed as positive and negative based on the manufacturer’s recommended cutoff value (Table-1).

**Table 1:** ELISA kit used for the detection of specific antibody with cutoff value.

Agent^a^	ELISA test^b^	Positive threshold^c^
PCV2	Ingezim Circo IgG (INGENASA)	O.D negative control+0.25
CSFV	HERDCHEK CSFV Ab (IDEXX)	blocking % ≥40%
PRRS	HERDCHEK PRRS 3X Ab (IDEXX)	S/P≥0.4

a PCV2=*Porcine circovorus* 2, CSFV=Classical swine fever virus, PRRS=Porcine reproductive and respiratory syndrome virus.

b ELISA=Enzymelinked immunosorbent assay, IgG=Immunoglobulin G.

c O.D=Optical density, S/P=Sample/Positive

### Statistical analysis

Chi-square test at two degrees of freedom (5%) for detection of an association between prevalence of specific antibody with the year of collection and detection of the samples was performed in IBM SPSS statistics version 19 (SPSS, Inc., an IBM company, USA).

## Results and Discussion

Serum samples were collected randomly from different organized farms of the three hill divisions of Meghalaya. Farms were located in various agro-climatic zones. The samples were basically cohort samples that were collected from the Disease Surveillance Programme with an objective to access the prevalence of the most vulnerable swine pig diseases in this area. Antibodies to PCV2 and CSFV were detected in pig population in these farms from October 2014 to December 2016. Apart from PCV2 and CSFV, PRRS seroprevalence study was carried out routinely after samples collection to find out initial information regarding seroprevalence against the three commonly encountered porcine diseases of northeast India. Antibody prevalence varied among pathogens and time period. The highest prevalence during the selected time periods was detected for PCV2 (80.8% in 2014, 79.1% in 2015, and 96.2% in 2016) followed by CSFV (76.4% in 2014, 66.09% in 2015, and 25.5% in 2016) and PRRS (2.8% in 2014, 2.7% in 2015, and 3.62% in 2016) summarized in Table-2.

**Table 2:** Seroprevalence of PCV2, CSFV, and PRRS antibodies in serum samples of pig population in organized farms of Meghalaya as assessed by ELISA.

Time of collection	Viral pathogen	Number of samples collected	Number of samples tested	Positive samples	Percent positivity
2014	PCV2	500	500	404	80.8
	CSFV			382	76.4
	PRRS			14	2.8
2015	PCV2	292	292	231	79.1
	CSFV			193	66.09
	PRRS			8	2.7
2016	PCV2	1106	1106	1064	96.2
	CSFV			282	25.5
	PRRS			40	3.62

PCV2=*Porcine circovorus* 2, CSFV=Classical swine fever virus, PRRS=Porcine reproductive and respiratory syndrome virus, ELISA=Enzymelinked immunosorbent assay

In this study, the overall prevalence of PCV2 antibodies detected in 2014-2016 time period was 89.51% which is somewhat indicating a higher incidence rate when compared to earlier reports where 78.24% PCV2 seroprevalence was detected in 2011-2013 time period [25]. The high seroprevalence of PCV2 specific antibodies in both the time period indicates that the virus is persistent in farm animals with or without any clinical symptoms which may be due to the prolonged incubation time and asymptomatic nature of the virus. Thus, the immunosuppression induced by PCV2 infection predisposes pigs to the coinfecting agents or if the coinfections promote PCV2 infection and the development of Porcine Circovirus Associated diseases (PCVAD).

In case of CSFV infection, the overall seroprevalence observed in this study was 45.15% which is comparatively very less compared to earlier reports where 90.9% CSFV seroprevalence was detected [26]. However, this report may not contradict the present study as the sample size was very less. In India, CSFV has not been studied systematically, although genomic and phylogenetic study was reported almost throughout the country, the epidemiology of the disease is largely unknown [27]. Interestingly, the animals positive for CSFV antibody are also positive for PCV2 antibody, i.e., 45.15% are found to have combined infection of PCV2 and CSFV.

Detection of virus-specific antibody in animals indicates an indirect evidence of virus persistence in the farms. This implies that the swine population acts as a major carrier of the viruses and thus plays an important role in its dissemination. Harboring these viral pathogens without showing any distinguishable symptoms, these animals may be considered as reservoir host as the viremia is prolonged and most of the infections are subclinical.

The samples were collected from suspected and healthy pigs without any noticeable clinical symptoms. Infection of PCV-2 in a farm often represents subclinical infection, in which pigs may act as potential virus shedders[6]. PCV2 being ubiquitous can be detected in almost all small herds to big farms. Studies based on herds revealed that PCV2 is shed in a similar amount by nasal, oral, and fecal route [28] from affected animals to healthy animals in close proximity in same pen or neighboring pens. In some experimental studies, the airborne transmission was proved to be another aspect for PWMS transmission [29]. Therefore, it is necessary to observe PCV-2 infection in a farm either serologically or by PCR to prevent the pigs from PMWS and minimize economic losses. However, even though PCV-2 is a principal causative agent of PMWS, several other microbial pathogens can act as cofactors for this disease [30]. Huang *et al*. [9] reported that PCV2 being immunosuppressive in nature may be considered as the reason to decrease the efficacy of the lapinized CSFV vaccine used in some farms. The present study indicates the same, as the organized farms where CSFV vaccination is a regular practice failed to provide considerable CSFV antibody titer (data not shown). The seroprevalence of CSFV in rural areas was found to be 74.7% (separate manuscript). The CSFV seroprevalence of 45.15% in farm animals (in this study) was comparatively less compared to that of rural areas despite CSFV vaccination history in some farms with lapinized vaccine due to unavailability of the vaccine. The most probable reason of vaccine failure may be due to an inappropriate titer of the vaccine, cold chain abuse, and inappropriate vaccine dose or may be concurrent PCV-2 infection that prevents the post-vaccination titer of antibody to reach optimal levels which support that CSF is endemic in India [26].

Samples were collected randomly from an organized farming system which comprises weaned piglets of 40-45 days and growers mostly. Samples collected from sows showed equal impact as growers on the ground of seroconversion and distribution of the viruses, which could be a signal about the persistence of PCV2 that could possibly dominate over CSF in co-infected animals. The presence of PCV2 can also have an effect on the vaccination efficacy against CSF thus leading to vaccination failures due to PCV immune suppression. An interesting feature of coinfection would be to investigate as to how virus divergence and recombination could progress and the evolutionary rates of both viruses need to be analyzed.

Most of the samples included in this study are from the piglets group, and the disease status of the animals reflects the incidence of diseases more compared to any post-vaccination seroconversion. This study reveals an alarmingly high proportion of PCV2 and CSFV antibodies in all the farms (included in the study) which confirms the epidemicity of the diseases and an alarming indication of disease outbreaks. Compare to PCV2 and CSFV prevalence, the prevalence of PRRS antibodies is very less. The incidence of PRRS antibody has decreased over the years since 2011 (data not shown) when PRRS was first reported from Animal Health Division, ICAR, Meghalaya and still the referral lab for PRRS diagnosis in North East India. The low antibody prevalence of PRRS in farm animals can be a boom as PRRS acts synergistically with PCV2 to induce PCVAD [31,32].

## Conclusion

There are few reports regarding PCV2 infections/outbreaks in pigs associated with reproductive failure from northern and southern part of India [5,7] but till date; there are no reports regarding concomitant infection of CSFV and PCV2 from India. In India, vaccination against PCV2 is not practiced. PCV2 seems to be ubiquitous in pigs [33], but the infection status of newborn piglets depends on the day of gestation when infection occurs. The high PCV2 seroprevalence and the reduced efficacy of CSFV lapinized vaccine indicate the ubiquitous nature of the virus in this study. To the best of our knowledge, this is the first report of coinfection of CSFV and PCV2 where a number of stillbirths, mummification, premature deliveries, and abortion cases were encountered. Common conclusion is that post CSFV vaccination outbreaks are mainly due to an inappropriate titer of the vaccine, cold chain abuses, and inappropriate vaccine dosing.

The study had able to resent the endemicity of CSFV in organized farms of Meghalaya, which is retarding the growth of swine industry in the state; simultaneously it had shown the presence of hidden threat of PCV-2 which is silently affecting the pig population of the region. PRRS one of the important transboundary disease which had created havoc in Mizoram [34,35]; the neighbor state of Meghalaya and had earlier shown its presence in the few pockets of this state is also present. PRRS was well controlled in the state due to proper control measure such as maintenance of farm hygiene, seromonitoring, and restricted animal movement from affected farms and culling of infected animals but regular seromonitoring should be prioritised even now so as to check this dreaded diseases incorrect time otherwise it has huge potential to increase the disease burden to the pig industry of the state.

## Authors’ Contributions

PR, SH, and AKC formed the study area and carried out the experiments. AK helped in sample collection and documentation. The US contributed in statistical framework and analysis. AS and IS contributed information of study design, intellectual inputs and manuscript checking and review. All authors participated in the scientific discussion. All authors read and approved the final manuscript.
